# Nanocellulose-Based Materials for Water Purification

**DOI:** 10.3390/nano7030057

**Published:** 2017-03-05

**Authors:** Hugo Voisin, Lennart Bergström, Peng Liu, Aji P. Mathew

**Affiliations:** Department of Materials and Environmental Chemistry, Stockholm University, SE-10691 Stockholm, Sweden; hugo.voisin@mmk.su.se (H.V.); peng.liu@mmk.su.se (P.L.)

**Keywords:** cellulose nanofibers, cellulose nanocrystals, membranes, water purification, adsorption

## Abstract

Nanocellulose is a renewable material that combines a high surface area with high strength, chemical inertness, and versatile surface chemistry. In this review, we will briefly describe how nanocellulose is produced, and present—in particular, how nanocellulose and its surface modified versions affects the adsorption behavior of important water pollutants, e.g., heavy metal species, dyes, microbes, and organic molecules. The processing of nanocellulose-based membranes and filters for water purification will be described in detail, and the uptake capacity, selectivity, and removal efficiency will also be discussed. The processing and performance of nanocellulose-based membranes, which combine a high removal efficiency with anti-fouling properties, will be highlighted.

## 1. Introduction

**Figure 1:**
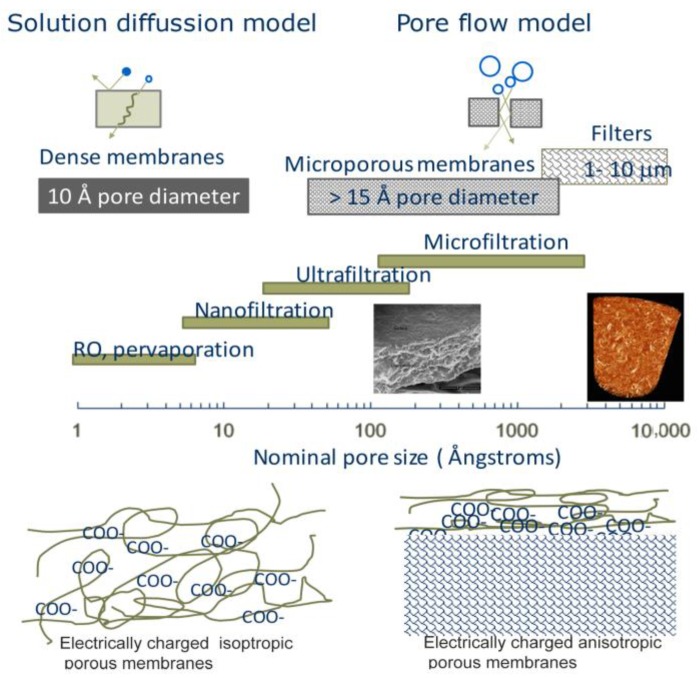
Schematic illustration highlighting the different types of membranes and filters. A scanning electron microscope (SEM) image of a nanocellulose membrane and X-ray tomograph of the nanocellulosic 3D porous structures (filter) are included, together with illustrations (below) of charged membranes (based on Reference [1]).

Membranes and filters can be used to separate different chemical species by allowing some species to pass while other are stopped [1,2]. The selectivity of a membrane is related to the microstructure and chemistry of the membrane material. Membranes can be designed with well-define pores for size exclusion and a tailored surface chemistry in order to selectively adsorb specific solutes [1,3,4]. Figure 1 shows some common membrane and filter configurations, and also summarizes the membrane classes based on pore size.

**Figure 2:**
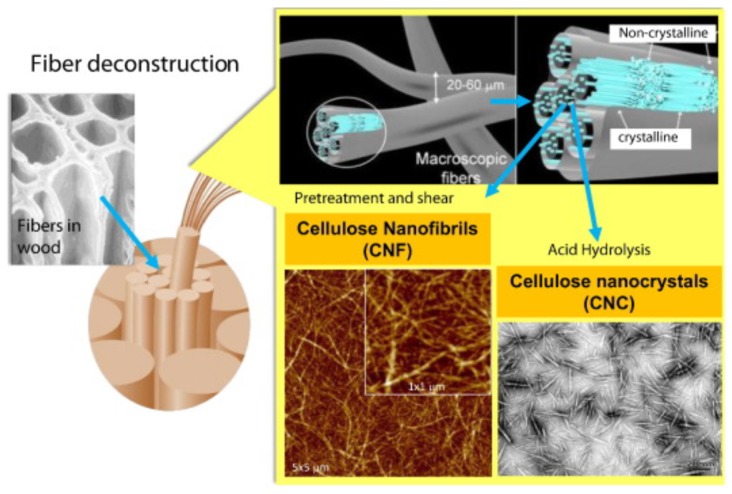
Schematic illustration of cellulose nanofibrils (CNF) and Cellulose nanocrystals (CNC) production from fiber cell walls by mechanical and chemical treatments (adapted with permission from [8]).

Cellulose nanocrystals (CNC) and cellulose nanofibrils (CNF) (Figure 2) are rod-like nanoparticles, with lengths varying between 100 and 2000 nm, and diameters ranging between 2 and 20 nm, depending on the preparation route and origin of the cellulose. Preparation processes usually involve acid hydrolysis using hydrochloric or sulfuric acid. Sulfuric acid reacts with the cellulose surface hydroxyl groups to form sulfate halfesters, which lead to negatively charged, and thus electrostatically stabilized, CNC nanoparticles. The use of hydrochloric acid results in unmodified surface hydroxyl groups and thus generates CNC particles with a very low surface charge density, which are prone to flocculation. The interested reader is referred to reviews by Habibi et al. [5,6] for more information of the properties, uses, and preparation routes of CNC. An exhaustive list of nanocellulose chemical modification routes can also be found [6]. Bacterial cellulose (BC) is a biosynthesized nanocellulose with a high degree of crystallinity [7].

The combination of high strength, chemical inertness, hydrophilic surface chemistry, and high surface area makes nanocellulose a very promising material for high-performance membranes and filters, in order to selectively remove contaminants from industrial and drinking waters. The high mechanical strength and rigidity of nanocellulose [9,10] is important in high-pressure, water-treatment applications. Nanocellulose with a high degree of crystallinity is chemically inert in aqueous media [11], except at very high pH-values [8]. The intrinsic hydrophilicity of nanocellulose is expected to reduce bio-fouling and organic fouling [12].

Defibrillation of cellulose fiber into nanocellulose results in a drastic increase of the available surface area. Depending on the preparation method, the specific surface area of nanocellulose can approach 500 m^2^/g [13,14], while the specific surface area of soft cellulose pulp typically ranges between 1 and 4 m^2^/g [15]. This increase in surface area is related to an increase in the availability of the hydroxyl groups on the surface of nanocellulose, where functional groups or molecules can be grafted using, for example, carboxylation, sulfonation, (2,2,6,6-tetramethylpiperidin-1-yl)oxyl (TEMPO)-mediated oxidation, phosphorylation, esterification, etherification, silyation, and amidation [6,16,17,18,19]. Indeed, previous work has demonstrated how carboxyl groups, sulfonate groups, and phosphonate groups can be grafted onto nanocellulose for the selective uptake of contaminants [20] in water remediation [17,21,22,23]. Nanocellulose-based membranes and filters have been shown to remove metal ions [4,24,25], dyes [26,27,28], metal [28,29,30] and microbes [28,30]. Organic contaminants, such as oils and cyclohexenes, have also been removed using a modified nanocellulose matrix grafted with hydrophobic or oleophilic functionalities [31,32].

This review will focus on the use of nanocellulose-based materials for the uptake and removal of pollutants from water. The major biosorption and adsorption mechanisms will be briefly described, followed by a detailed description of the uptake of heavy metals and organic pollutants from aqueous solutions by functionalized nanocellulose. This review is only concerned with nanocellulose; readers interested in the sorption properties of cellulose fibers and regenerated cellulose are recommended to consult recent reviews on those systems [33,34]. Processing routes to produce nanocellulose-based membranes with tailored pore sizes and mechanical stability are described in detailed, and the structural and chemical properties are related to the rejection rate, adsorption capacity, and selectivity of the membranes.

## 2. Sorption of Pollutants by Functionalized Nanocellulose

### 2.1. Biosorption and Adsorption Mechanisms

The basic principle of adsorption is the binding of a chemical species to a sorbent. The adsorption process is controlled by the interactions between the sorbent and the sorbate, and is often modeled with adsorption isotherms, which describe the relationship between the surface concentration of the adsorbate and the adsorbent bulk concentration at a constant temperature. The most widely-used isotherm for sorption on cellulose is the Langmuir isotherm [35], initially developed to describe the adsorption of gases on various surfaces. The Langmuir isotherm can be expressed as Equation (1):

(1) $$q_{e}=\frac{Q_{max}bC_{e}}{1+bC_{e}}$$

where *C_e_* (mg/L) is the equilibrium concentration of the species (e.g., metal ion or organic pollutant) in the solution; *q_e_* (mg/g) is the (equilibrium) adsorbed amount at this solution concentration; *Q_max_* (mg/g) is the maximum adsorption capacity per gram of sorbent; and *b* (L/mg) is the Langmuir constant related to the adsorption energy of the system. The values of *Q_max_* and *b* are generally obtained by plotting the experimental data in a linearized form of the Langmuir equation. The maximum uptake and adsorption energy can then be used to compare the performances of various adsorbents for specific adsorbates.

The Freundlich isotherm, an empirical model, is sometimes used to model the adsorption behavior. This model takes into account multiple adsorbed layers and heterogeneous site energies. Its general form Equation (2) is:

(2) $$q_{eq}=K_{F}C_{eq}^{1/n}$$

where K_F_ and *n* are the Freundlich constants related to the adsorption capacity and adsorption intensity, respectively [36]. The Langmuir and Freundlich models can be coupled together if, for example, there is competition between different adsorbates which is generally the case in wastewaters. The majority of the reviewed studies presented here that modeled their data have used either the Freundlich or the Langmuir isotherms.

Biosorption represents a specific type of sorption in which the sorbent is of biological origin, e.g., microorganisms or biopolymers. The capacity of biosorbents, from various origins, to adsorb (or absorb) and release contaminants make them useful for water purification. Indeed, large volumes of biomass are available from waste or naturally-occurring organisms, such as plants, algae, or fungi [37]. Various microorganisms are also able to uptake organic pollutants [38]; biosorption is a versatile solution for soil remediation and water purification [18]. The modelization of biosorption mechanism is complicated, however, by the complexity of both the biosorbent and its varied active sites and of the complex solution chemistry of metallic compounds. The previously described empirical models do not reflect physical reality, and they usually cannot be extrapolated to predict biosorbent performances under different conditions. They are still useful to compare adsorption capacity in controlled conditions. The same observation can be done for the modelization of biosorption kinetics (with the most widely used models being the first-order and the pseudo second order). Kinetic studies gives very important information for biosorption process design but are currently insufficiently presented in the literature.

### 2.2. Uptake and Removal of Heavy Metal Species from Water

Heavy metal species are highly toxic pollutants in water that can originate from either industrial or natural sources. Heavy metals can exist under different forms in water and form both anionic and cationic species. Efficient uptake of heavy metal species usually requires that the dominating species be identified, and that the surface chemistry of the sorbent material be optimized to maximize the uptake capacity, partitioning coefficient, and selectivity. The solution pH is an important parameter that can have a significant influence on uptake and selectivity [39]. Liu et al. reported that cellulose nanocrystals (CNC) obtained by sulfuric acid hydrolysis have a higher Ag(I) adsorption capacity (34 mg/g) than cellulose nanofibrils (CNF) obtained through mechanical grinding (14 mg/g), due to the sulfate (–SO_3_^−^) functional groups on the surface of CNC. The adsorption behavior was found to be pH dependent, and the best adsorption performance was found to be near neutral pH; the uptake is significantly lower under acidic pH conditions, as H^+^ competes with Ag^+^ for the –SO_3_^−^ functional groups on the nanocellulose surface [22].

**Figure 3:**
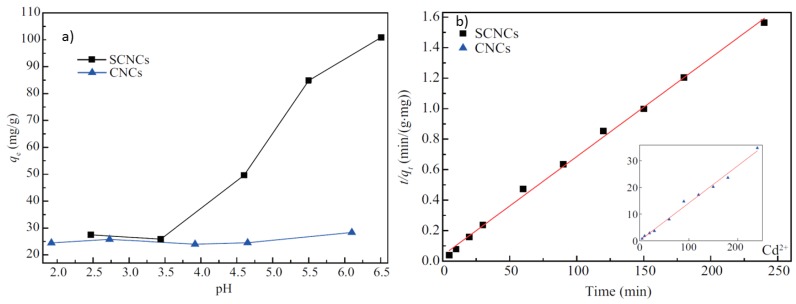
Comparison of adsorption behaviors of sulfated CNCs (SCNS) and carboxylated CNCs; (**a**) pH dependency of CNC adsorption on Cd^2+^; (**b**) plot of *t*/*q^t^* (where *q_t_* (mg/g) is the amount of metal adsorbed at time (*t*) as a function of time). The linearity of SCNCs correspond to a good fit by the pseudo second order kinetic model. CNCs: Sulfated CNCs; SCNCs: Carboxylated CNC after reaction with succinic anhydride. Adapted with permission from [42].

Seminal work by Isogai and coworkers has shown that C6 carboxylate groups can be selectively formed on the surface of cellulose nanofibers using TEMPO-mediated oxidation (TOCNF) [17]. TOCNF has been used to remove Pb(II), La(III), and Ag(I) ions from aqueous solutions [40]. Sehaqui et al. [14] performed a systematic study on the adsorption of Cu(II) onto TOCNF as a function of pH and carboxylate group content on the fibril surface. The study showed that the Cu(II) adsorption onto nanofibers increased linearly with carboxylate content for pH values between 3 and 7. The adsorbed Cu(II) ions could be desorbed from the nanofibers through acidic washing, which suggest that TOCNF can be regenerated easily [14,21]. Ma et al. [41] showed that TOCNF could also adsorb up to 167 mg/g of UO_2_^2+^ at pH = 6.5. Yu et al. [42] reported that carboxylation through esterification of hydroxyl surface groups onto CNC with succinic anhydrid resulted in a sorbent with a very high maximum adsorption uptake of Pb(II) (458 mg/g) at pH = 5.5 and Cd(II) (335 mg/g) at pH = 6.5. The kinetics of the Pb(II) and Cd(II) adsorption process on CNC were very fast (maximum uptake was obtained within 5 min or less) and could be well fitted by the pseudo second-order kinetic model; The Langmuir adsorption isotherm fit well with the equilibrium adsorption data (Figure 3).

The adsorption process, which follows the pseudo second-order kinetic model, indicates that the chemisorption process is the rate determining step, in contrast to first order kinetic models, where the diffusion of species to the adsorbent is the rate determining step. The maximum metal ion adsorption of succinic-anhydride-modified mercerized nanocellulose ranged from 42.3 mg/g to 218 mg/g, and was successfully regenerated using ultrasonic treatment [43]. Carboxylic groups can also be introduced by, first, selectively oxidizing the C2 and C3 hydroxyl groups, and then by oxidizing the aldehyde groups to form 2,3-dicarboxyl groups in aqueous acid medium [44]. Sheikhi et al. [45] showed that CNC functionalized following this route can display a maximum uptake capacity for Cu^2+^ of 185 mg/g at pH = 4. It should be noted that the maximum uptake of metal ions of carboxylated CNCs is usually much higher than the uptake of non-modified CNFs obtained through mechanical treatment (11 mg/g for Cd^2+^, 10 mg/g for Pb^2+^, 11 mg/g for Ni^2+^ at pH = 6.5) [46], which further demonstrates the importance of surface functionalization in order to maximize the uptake capacity of metal ions from aqueous solutions. It has been described that amino-functionalized bacterial cellulose [47], prepared by reaction with epichlorhydrin and diethylenetramine, can adsorb cations through coordination interaction, with a maximum adsorption uptake capacity of Cu^2+^ and Pb^2+^ at pH = 4.5 of 63 mg/g and 87 mg/g, respectively. The intake of this system dramatically decreases when the pH is lowered due to the protonation of amine groups.

The adsorption process, which follows the pseudo second-order kinetic model, indicates that the chemisorption process is the rate determining step, in contrast to first order kinetic models, where the diffusion of species to the adsorbent is the rate determining step. The maximum metal ion adsorption of succinic-anhydride-modified mercerized nanocellulose ranged from 42.3 mg/g to 218 mg/g, and was successfully regenerated using ultrasonic treatment [43]. Carboxylic groups can also be introduced by, first, selectively oxidizing the C2 and C3 hydroxyl groups, and then by oxidizing the aldehyde groups to form 2,3-dicarboxyl groups in aqueous acid medium [44]. Sheikhi et al. [45] showed that CNC functionalized following this route can display a maximum uptake capacity for Cu^2+^ of 185 mg/g at pH = 4. It should be noted that the maximum uptake of metal ions of carboxylated CNCs is usually much higher than the uptake of non-modified CNFs obtained through mechanical treatment (11 mg/g for Cd^2+^, 10 mg/g for Pb^2+^, 11 mg/g for Ni^2+^ at pH = 6.5) [46], which further demonstrates the importance of surface functionalization in order to maximize the uptake capacity of metal ions from aqueous solutions. It has been described that amino-functionalized bacterial cellulose [47], prepared by reaction with epichlorhydrin and diethylenetramine, can adsorb cations through coordination interaction, with a maximum adsorption uptake capacity of Cu^2+^ and Pb^2+^ at pH = 4.5 of 63 mg/g and 87 mg/g, respectively. The intake of this system dramatically decreases when the pH is lowered due to the protonation of amine groups.

**Figure 4:**
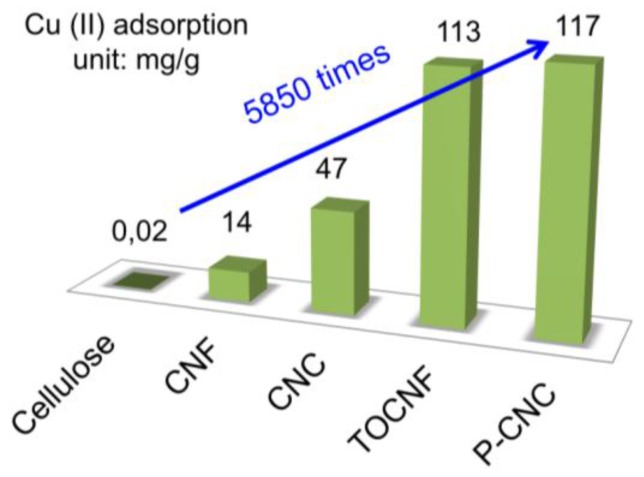
Comparison of Cu(II) adsorption capacities among cellulose, nanocellulose and surface modified nanocellulose [14,48,50]. Comparison of cellulose with cellulose nanofibers (CNF), cellulose nanocrystals (CNC), tempo oxidized cellulose nanofibers (TOCNF), and phosphoryl cellulose nanocrystals (P-CNC) are given.

Liu et al. [48] reported that enzymatically-phosphorylated cellulose nanocrystals and nanofibers have a high maximum uptake capacity for Ag(I), Cu(II), and Fe(III) at pH = 4 of 136 mg/g, 117 mg/g, 115 mg/g (for CNC), and 120 mg/g, 114 mg/g, and 73 mg/g (for CNF), respectively. Figure 4 shows the dramatic enhancement of Cu(II) uptake capacity (5850 times) after disintegration of cellulose fibers into their nanosized constituents, and their surface phosphorylation.

Xanthated nanocellulose prepared by steam explosion of banana fiber, followed by acid hydrolysis and xanthation, displayed a maximum uptake capacity of 154 mg/g of Cd(II) at pH = 6 [49].

Sorption of anionic metallic species, such as arsenates or chromates, from aqueous solution can be achieved if nanocellulose is functionalized with positively charged groups. Singh et al. [19] reported that aminated CNC, prepared using sodium periodate oxidation, can adsorb 98% of dichromate ions in a 25 mg/mL solution at pH = 2.5. Positively-charged CNC can also be prepared from sulfated CNC by reaction with epoxypropyltrimethylammonium chloride (EPTMAC) [51]. Biphosphonated nanocellulose obtained through successive oxidation with sodium periodate, and reaction with sodium alendronate [52], could efficiently remove vanadium in its metavanadate (VO_3_^−^) form with a maximum adsorption capacity of 194 mg/g at pH = 2.

Polymers with chelating or ionic functionalities have been grafted onto cellulose [33], but similar studies on nanocellulose are sparse. [6] Amino-terminated polymers, which displayed a maximum uptake capacity of mercury on cellulose of 288 mg/g at pH = 7 [53], have been grafted onto TEMPO carboxylated CNCs using carbodiimide-catalyzed amidation in water [54]. Amide-bearing polymers, which have been shown to display a very high uptake of mercury when grafted onto cotton cellulose [55] (maximum uptake of 710 mg/g at pH = 6.2), have been grafted onto sulfated CNCs via radical polymerization [56]. Polymers with carboxylic acid functions have been grafted onto different sources of cellulose [57,58] which exhibited uptake capacity for various cations. Polylactic acid (PLA) has been grafted onto CNCs [59,60,61], and Zhang et al. [62] functionalized the surface of CNFs from bamboo through grafting of (poly)acrylic acid, and used the modified CNFs to remove Cu(II) from aqueous solutions. The adsorption capacity of modified bamboo CNFs was three times higher than that of pristine bamboo CNFs [62]. Zhang et al. [63] described that TOCNFs functionalized with polyethyleneimine (PEI) via crosslinking with glutaraldehyde displayed a higher uptake of Cu(II) at pH = 5 compared to PEI-grafted cellulose [64]. Carboxymethyl cellulose (CMC), a water-soluble derivative of cellulose, presents chelating carboxyl groups and can be grafted onto nanocellulose. Chen et al. [65] elaborated CNF-CMC aerogels, exhibiting adsorption capabilities for Ag^+^, Cu^2+^, Pb^2+^ and Hg^2+^ of 106 mg/g, 74.8 mg/g, 111.5 mg/g, and 131.4 mg/g, respectively. Other than grafting polymer, one can also use hybrid nanomaterials from nanocellulose to improve its adsorption capacity. Through reaction of microfibrillated cellulose with an organosilane containing an amine group, aminopropyltriethoxysilane (APTES), Hokkanen et al. [66] were able to obtain such a material, presenting at pH = 4.5, with maximum uptake capacities of 179 mg/g, 163 mg/g, and 388 mg/g for Ni^2+^, Cu^2+^, and Cd^2+^, respectively. This amino-functionalized system presents an adsorption maximum around pH = 4.5 and a lower adsorption capacity at higher and lower pH levels.

Table 1 present an overview of the adsorption properties of heavy metals of functionalized nanocellulose, and, when possible, a comparison with functionalized cellulose materials.

#### Removal of Organic Pollutants

Organic pollutants in water represent a very wide range of compounds and include dyes, oils, pesticides, and pharmaceuticals products. This review will be limited to the uptake of dyes and drugs by functionalized nanocellulose.

Organic dye pollutants generated by, e.g., the textile industry, pose a significant environmental problem in many parts of the world. Organic dyes usually possess a complex aromatic structure and can display cationic, anionic, or non-ionic properties. Their interactions with cellulose have been crucially important over human history, and associated mechanisms have recently been described in detail [69]. Carboxylated nanocellulose has been extensively studied for the sorption of cationic dyes. Carboxylated CNCs with a high COOH content of 2.1 mmol/g, prepared using TEMPO-mediated oxidation, were found to result in a significantly higher uptake (769 mg/g at pH = 9) of the cationic dye methylene blue, compared to CNCs with sulfate groups on their surfaces (118 mg/g at pH = 9) [70]. CNCs with carboxylic acid functionality (carboxyl content of 3.4 mmol/g), prepared by esterification of surface hydroxyl groups with maleic anhydride [71], displayed a high uptake capacity for several cationic dyes, e.g., crystal violet, methylene blue, malachite green, and basic fuchsin, with a maximum crystal violet uptake of 244 mg/g at pH = 6. Carboxylated CNCs prepared by one-step oxidation [72] using ammonium persulfate showed an adsorption capacity of 101 mg/g for methylene blue, at a neutral pH. [73] Carboxylated CNCs prepared from microcrystalline cellulose by extraction, using a mixture of hydrochloric and citric acid [74], could adsorb methylene blue with an uptake that is directly related to the quantity of carboxyl groups. Previous studies show that carboxylated CNCs display a pH dependency for cationic dye adsorption that are similar to those previously described for cationic metallic species, i.e., a decrease of the maximum uptake at a low pH (<3) due to the protonation of carboxylic functional groups.

Anionic dyes are usually removed using nanocellulose with cationic functionality, e.g., amino-functionalized CNC. Cationic CNCs prepared by successive sodium periodate oxidation, followed by reaction with ethylenediamine [27], present a maximum uptake of 556 mg/g of acid red GR. The pH dependency of the adsorption capacity for positively-charged CNCs depends on the pH-dependent dissociation of functional groups. Amine functional groups usually result in a maximum adsorption at low pH and a significant decrease in the uptake capacity at pH values of 8 and higher. Kinetic uptake studies on cationic CNFs obtained through quaternization with glycidyltrimethylammonium chloride (EPTMAC) [75] showed an uptake of up to 664 mg/g and 683 mg/g of Congo red and acid green 25, respectively, in less than a minute. CNCs can also be used to form crosslinked microgels with polyvinylamine (PVAm) that exhibit a high affinity for both cationic and anionic dyes, with maximum adsorption uptakes for acid red GR, Congo red 4BS, and reactive light yellow K-4G, of 896 mg/g, 1469 mg/g, and 1250 mg/g, respectively [76]. The same functionalization route was also studied by Zhu et al. [77] on dialdehyde functionalized cellulose powder, but using hyper-branched polyethyleneimine, which presented a higher Congo red adsorption (2100 mg/g) and a high cationic basic yellow adsorption (1860 mg/g).

**Figure 5:**
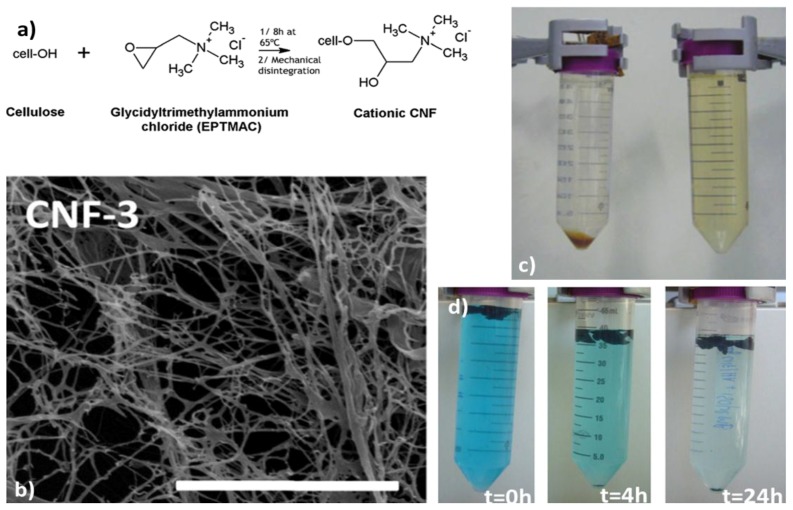
(**a**) Reaction scheme of the cationic functionalization of cellulose nanofibers (CNF); (**b**) SEM micrographs of the cationic CNF; scale bar is 4 µm; (**c**) photograph showing (**right**) a brown solution of humic acid at 13 mg/L, and (**left**) the adsorption of humic acid by cationic CNF (sediment); (**d**) Color change as a function of exposure time due to methylene blue uptake by the CNF/humic acid system. Adapted with permission from [78].

Cationic CNFs are efficient adsorbents of negatively-charged water contaminants, such as nitrate, phosphate, fluoride, and sulfate. As expected, the adsorption capacities of all the anions increased with the surface charge density of CNFs. Cationic CNFs displayed a higher selectivity toward multivalent ions (PO_4_^3−^ and SO_4_^2−^) than monovalent ions (F^−^ and NO_3_^−^) [23]. Figure 5 shows that cationic CNFs obtained through reaction of cellulose with EPTMAC, and subsequent mechanical disintegration, displayed a high and fast uptake of humic acid, a common organic pollutant [78]. The humic acid adsorption capacity (310 mg/mg) observed stands amongst the highest capacities reported in the literature. Faster adsorption kinetics were observed at low pH values, which was attributed to the coiled conformation of humic acid in this pH range, allowing a thicker humic acid layer to be adsorbed onto CNFs.

Pharmaceutical molecules, such as antibiotics, which end up in waste water, is of growing concern, as they can accumulate in aquatic organisms [79]. There are extensive reports on the use of nanocellulose as drug carriers, but studies on the adsorption of drugs onto nanocellulose are sparse. Jackson et al. [80] first confirmed the capacity of sulfated CNCs to adsorb ionized drugs, such as doxorubicin hydrochloride (DOX) and TC in water. The composition of the solvent (phosphate buffer saline (PBS) or deionized water) demonstrated that there is a competition between ions and ionic drugs, e.g., DOX. Hydrophobic drugs could also be adsorbed after grafting a surfactant, e.g., cetyl trimethylammonium bromide, onto CNCs in order to hydrophobize them, with a maximum uptake of around 0.1 mg/g for docetaxel and paclitaxel. Other routes for the hydrophobization of nanocellulose surfaces include reaction with alkyl isocyanate [81]. Rathod et al. [82] recently reported that sulfated CNCs can be used to adsorb one of the most used antibiotics, tetracycline hydrochloride (TC), from water; maximum sorption uptake being approximately 7 mg/g at pH = 5. The pseudo second-order kinetic model fit reasonably well with the adsorption profile, indicating that the biosorption rate is mainly controlled by chemisorption. Carboxylated CNCs obtained via TEMPO-mediated oxidation have presented comparable binding capacities of cationic drugs [83]. Despite the relatively low amount of research done on nanocellulose–pharmaceutical molecules, it is clear that nanocellulose can be an efficient adsorbent of drugs, similar to the much more documented field of dye adsorption.

The adsorption capacities for organic molecules of the different surface functionalization are summarized in Table 2.

**Figure 6:**
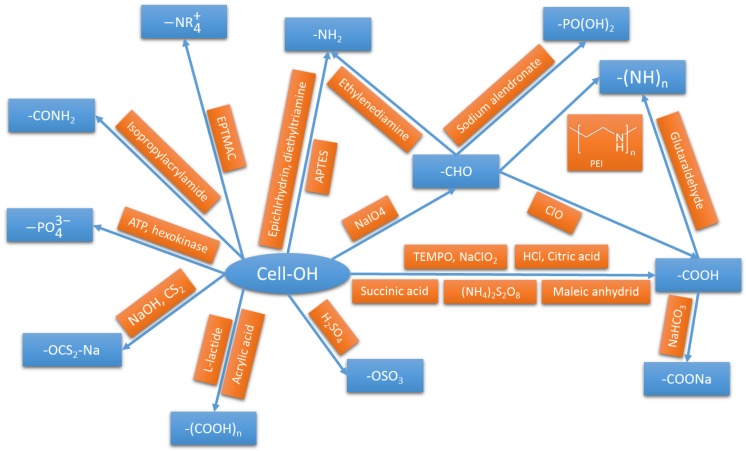
Summary of the main routes of surface functionalization of nanocellulose for the adsorption of pollutants.

We demonstrated here, using several examples, the versatility of nanocellulose as a bioadsorbent. Through various preparation routes and surface functionalizations (Figure 6), nanocellulose can be tailored to adsorb a wide range of pollutants.

The summary of the functionalization routes, and of their respective efficiencies, presented in Figure 6 and Table 1 and Table 2, highlights several advantages and disadvantages for each route. Concerning the sorption of cationic species using direct functionalization of the cellulose surface, carboxylation of nanocellulose is the most studied process. It displays some of the highest adsorption capacities for metallic cations. Moreover, it is usually obtained through a one-step procedure (esterification with dicarboxylic acid) or even during the preparation of nanocellulose (TEMPO-mediated oxidation). It also displays enhanced cationic dye adsorption compared to sulfated nanocellulose. Functionalization with amino-groups can lead to a very high adsorption capacity for various metallic cations. However, it exhibits an adsorption maximum at a given pH, with a decreased efficiency at lower and higher pH levels, when carboxylic groups efficiency decreased only under acidic conditions. It also displays good adsorptive properties for acidic dyes.

The grafting of polymers bearing reactive functions is a very promising technique to obtain highly-adsorptive cellulose for various species. Despite presenting relatively low performances for the adsorption of metallic cations on nanocellulose, the grafting of carboxylic-containing polymers onto cellulose of sawdust led to a high adsorption uptake, leaving room for a potential increase through reduction in sizes of systems. Nanocellulose grafted with polymer-containing amino groups exhibited a very high adsorption capacity for various types of dyes. A disadvantage that may present in polymer grafting routes over direct functionalization is the difficulty to integrate them into membranes for the elaboration of filtrating devices, due to the heavy modification of the nanocellulose aspects and dispersability.

## 3. Nanocellulose-Based Water Purification Membranes and Filters

**Figure 7:**
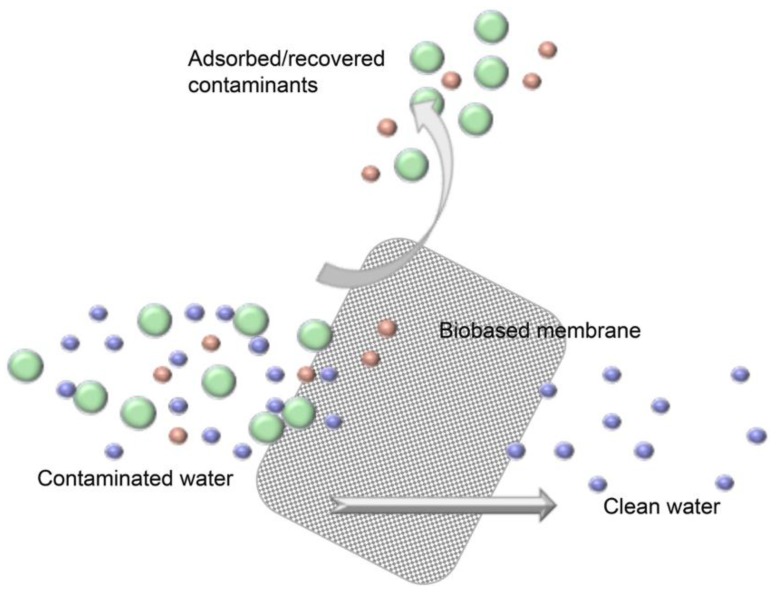
Schematic representation of nanocellulose-based membranes, where the nanocellulose-based membrane filter adsorbs and separates contaminants from polluted water.

Though generally termed as a membrane, nanocellulose-based materials may function as a membrane, filter, or adsorbent, depending on pore structure and mode of operation (static or cross-flow mode). The performance of nanocellulose-based membranes is mainly related to the capability of the high surface area nanocellulose fibers to selectively adsorb contaminants from water streams (Figure 7). The selective adsorption of specific contaminants is, as shown in the first part of this review, strongly related to the functional groups present on the surface of nanocellulose [20]. The inherent hydrophilicity of nanocellulose, combined with a controlled surface chemistry, have the potential to reduce bio-fouling and organic fouling, which are inherent problems in membrane technology [12].

### Processing and Properties of Nanocellulose-Based Water Purification Materials

The main challenge in the processing of membranes based on nanocellulose lies in designing membranes that provide optimal access to functional sites, combined with a high flux and the mechanical stability of membranes. Most relevant and frequent processing techniques for nanocellulose membranes found in the literature are (a) impregnation of electrospun mats with nanocellulose; (b) vacuum filtration and coating; and (c) freeze-drying, with or without a matrix phase. The processing routes, their structural characterizations, and water purification performances will be discussed in detail.

Impregnation of electrospun mats: The first example of nanocellulose-based membranes for water purification involved impregnation of the electrospun mats with functionalized nanocellulose [24,82,83]. Impregnation of electrospun mats with nanocrystals of different aspect ratios showed a possibility to process charged microporous membranes with isotropic or anisotropic structures, depending on the extent of impregnation in the electrospun layer [84,85,86].

This versatile technique has been used to produce mechanically-strong composite nanofibrous membranes by introducing TOCNC into an electrospun polyacrylonitrile (PAN) nanofibrous scaffold, and heating the composite to 100 °C for 10 min, in order to cross-link the cellulose nanocrystal (CNC) network [24]. The membrane was supported on a strong polyethylene terephthalate (PET) nonwoven substrate, and the pore diameter was in the range of 50 nm. The TOCNC-containing composite membranes possessed a high negative surface charge density, and displayed a high adsorption capacity of positively-charged species, such as crystal violet (CV) dye. CV dye has a molecular diameter of approximately 1.6 nm, and the maximum adsorption capacity could be correlated with the surface charge density of the membranes. The study showed that the nanocellulose-based MF membrane exhibited 16 times higher adsorption capacity against a positively-charged dye relative to a commercial nitrocellulose-based MF membrane.

Ma et al. [25,85] demonstrated that electrospun nanofibrous membranes containing CNCs could be used to efficiently remove two bacteria (i.e., *Brevundimonas diminuta* and *Escherichia coli*) and one virus model (i.e., bacteriophage MS2) at a relatively high flux and low pressure.

**Figure 8:**
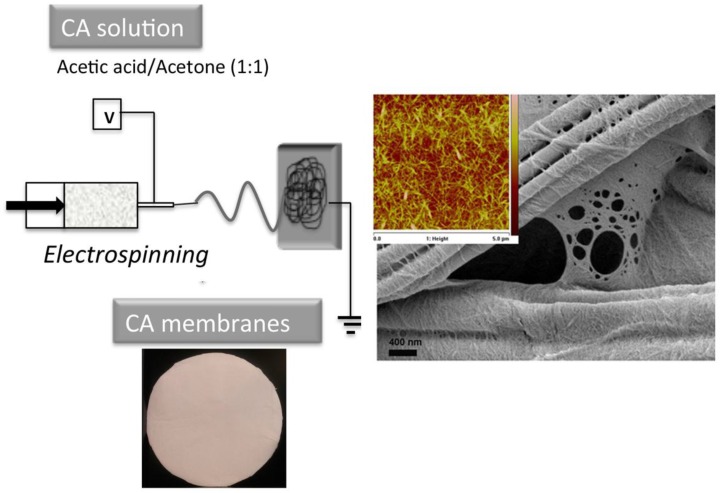
Processing of membranes via impregnation of cellulose acetate (CA) electrospun mats. Electrospinning process and the mats after impregnation with cellulose nanocrystals are shown. SEM image shows the electrospun mats coated with nanocrystals, and atomic force microscopy (AFM) images (inset) of the nanocrystals used for functionalization.

Goetz et al. followed a similar approach and developed fully bio-based membranes using cellulose acetate (CA) polymer-based electrospun mats as supports for chitin or cellulose nanocrystals, without an additional non-woven support layer [86]. The process resulted in CA fibers coated with nanocrystals with hydrophilic surfaces and a thin, microfiltration membrane with pore diameters in the range of 100 Å and a porosity of 85.6%. Figure 8 shows the processing route for CA membranes impregnated with cellulose nanocrystals, and an SEM image showing the electrospun mats coated with nanocrystals.

The CA-CNC membranes showed a higher flux (13,500 Lm^−2^·h^−1^·bar^−1^) than the PAN nanofiber mats impregnated with cellulose nanocrystals (5900 Lm^−2^·h^−1^·bar^−1^). This could be a result of larger pore sizes, lack of a support layer, and the higher hydrophilicity of the cellulose-acetate-based membranes in comparison to the PAN nanofiber-based membranes. The nanocrystal coating produced on the CA electrospun fibers created a very hydrophilic surface compared to the original hydrophobic CA mats. Recent work has shown that chitin nanocrystals can also be used to make CA membrane surfaces hydrophilic, with excellent antifouling properties [86].

Vacuum Filtration: Vacuum-filtration is a fast, simple, and scalable process to fabricate layered structures of cellulose nanomaterials. Fully bio-based membranes/adsorbents, based on nanocellulose, can be fabricated via vacuum filtration, followed by hot pressing, the so called ‘Nano paper approach’ [87,88]. This process results in a film/membrane, where the nanofibrils are densely packed to form a network with pore-dimensions in the range of the diameter of cellulose nanofibrils (3–5 nm). This opens up the possibility to control the pore sizes, and, accordingly, the presence and molecular weight cut-off of the resulting membranes by using nanocellulose with different fibril diameters. Membranes produced by the Nano paper method have been shown to effectively remove metal ions (Cu^2+^, Ag^+^, Fe^3+^), nitrates, fluorides, phosphates, sulfates, humic acid, and organic compounds [23,87,88,89].

Mautner et al. prepared nanofiltration membranes using vacuum filtration using nanocellulose fibrils with diameters in the range of 5–30 nm [88], which resulted in vacuum filtrated membranes displaying a small pore size, suitable for the rejection of molecules, with MW of 6–25 kDa; however, the flux was low (100 Lm^−2^·h^−1^·bar^−1^) [88]. Membranes produced by the vacuum filtration of TOCNF with diameters below 5 nm showed the combined capability of rejecting molecules of 6 kDa via size exclusion and rejection of Ca^2+^ ions [85]. Membranes produced using the Nano paper approach, using cellulose nanofibers with quaternary ammonium functionality, mixed with non-functionalized cellulose nanofibers, could be used to remove nitrates from a water stream [90]. The maximum flux of these membranes was 150 Lm^−2^·h^−1^·bar^−1^. It is clear that the major drawback of membranes produced by the Nano paper approach is their low presence (flux). Metreveli et al. showed that cladophora nanocellulose-based filter paper can act as a size-exclusion membrane for the removal of relatively large viruses, with sizes >50 nm [91].

Multilayered anisotropic membranes have been produced by vacuum filtering a thin layer of cellulose nanocrystals onto a support layer [92,93]. Multilayer membranes with a mixture of nanocelluloses have been produced by vacuum-filtration of cellulose nanofiber (CNF) suspensions, followed by dip coating the thin films into a dispersion of cellulose nanocrystals with sulfate or carboxyl surface groups [91]. This approach resulted in membranes with a narrow pore size of 74 Å. Post treatment with acetone increased the pore sizes to 194 Å, and the flux to 250 Lm^−2^·h^−1^·bar. These membranes showed a high removal of Ag^+^, Cu^2+^ and Fe^3+^ ions, suggesting that electrostatic adsorption and size exclusion both contribute to the high efficiency. The main drawback of this membrane was the low flux, which was attributed to the tight support layer.

In an attempt to address the issue of low permeability, anisotropic membranes were fabricated using cellulose microfibers as a support layer and cellulose nanocrystals with different surface functionalities (sulfate groups, carboxyl groups, phosphoryl group) in a gelatin matrix as a nanoporous top layer [90]. Bubble point measurements confirmed that the membrane pore size was in the microfiltration range of 5.0–6.1 μm, which provided very high water permeability (900–4000 Lh^−1^·m^−2^) at pressures of <1.5 bars. These membranes had good mechanical properties (tensile strength of 19 MPa) and these anisotropic, negatively-charged membranes successfully removed Ag(I) and Cu(II)/Fe(II)/Fe(III)) ions from mirror-making industry effluents. The sorption efficiency was highest for membranes consisting of CNCs with phosphoryl groups, followed by carboxyl and sulfated CNCs [90].

**Figure 9:**
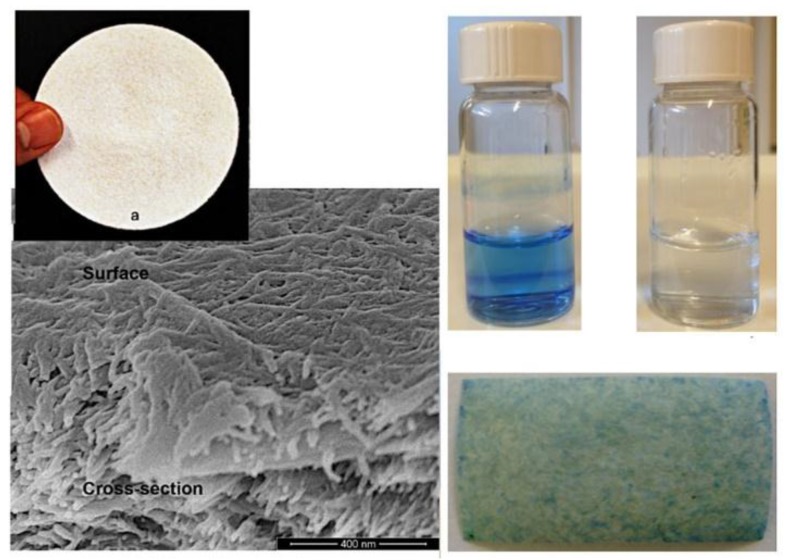
Visual appearance of freeze-dried membranes and SEM images showing the CNCs on the surface and bulk of the membrane. The membranes adsorbed 98% of Victoria blue 2B from water.

Freeze drying: Freeze drying, followed by compaction of a mixture of sulfated CNCs and chitosan [94], created microporous membranes with a thickness of 200 microns, capable of immobilizing positively-charged dye molecules. The pore diameters of the membranes were in the range of 10–13 nm, and the water flux was low (64 Lm^2^·h^−1^·MPa^−^^1^). Low mechanical stability was another drawback and is likely due to the absence of H-bonding after the freeze-drying process. Karim et al. [94] reported 84%, 69% and 98% removal rates for Methyl Violet 2B, Rhodamine 6G, and Victoria Blue 2B from an aqueous solution at pH 5 when using freeze-dried affinity membranes functionalized with sulfated cellulose nanocrystals (Figure 9).

## 4. Conclusions and Future Outlook

Nanocellulose is an efficient bioadsorbent that combines a high strength and flexibility with a high surface area and versatile surface chemistry. There are a wide range of facile hydrolysis and catalyzed process routes that produce nanocellulose into cellulose nanofibers and nanocrystals, with tailored surface properties. Nanocellulose with cationic or anionic surface groups have been shown to remove heavy metal pollutants from aqueous solutions. Nanocellulose with ionic and non-ionic surface groups have also been used to adsorb organic pollutants, e.g., dyes, oils, and pesticides, from aqueous solutions. We have given a detailed overview of the different functionalization routes of nanocellulose and related the surface properties to the adsorption capabilities and kinetics.

The surface functionality of nanocellulose combined with its ability to form mechanically stable flat sheets has been exploited to fabricate membranes for water purification. Vacuum filtration, different coating methods, and electrospinning are used extensively to produce membranes. The scaling up of the flat sheet membranes following a paper forming process using nanocellulose and the fabrication of spiral wound modules has been successfully demonstrated over the 2015–2016 period [95,96].

Nanocellulose-based membranes and filters are indeed very promising, but challenges related mainly to cost-effective upscaling, biofouling, and limitations in specificity need to be addressed. Recent efforts by several companies in the forest product area in the cost-effective and large-scale production of nanocellulose is expected to provide the market with large amounts of nanocellulose fibrils and crystals (e.g.,: Exilva, Borregaard Sarpsborg, Norway; CELISH/TIARA, Diacel FineChem Ltd., Tokyo, Japan; UPM Kymmene Ltd. Helsinki, Finland; StoraEnzo Ltd., Stockholm, Sweden; Celluforce, Quebec, Canada; MoRE Research,Örnsköldsvik, Sweden; Forest Products Lab., Madison, WI, USA). Developing cost-efficient processing routes of surface modified nanocellulose will be crucial for commercial implementation of nanocellulose-based membranes and filters. Indeed, cost- and energy-efficient processing and utilization of membranes as well as safe and cost-efficient disposal routes at the end of life are essential for market realization of this technology. Although modified nanocellulose often display uptake capacities that are comparable or even better than conventional adsorbents, e.g., activated carbon and ion exchange resins, there is a need to address and investigate the selectivity in the presence of more complex water streams. This may require the introduction of more specific and selective surface functionalities.

Approaches and methods used in the more mature polymer membrane field, e.g., breakthrough experiments, can provide more insight on the performance of nanocellulose-based membranes in real industrial applications and benchmark it against other water purification products. Research efforts to improve the understanding on membrane fouling, recycling, and regeneration need to be implemented.

The next phase of nanocellulose based water purification membrane research is expected to focus on hybrid membranes where nanocellulose is used in combination with other nanoparticles to improve pore structure, mechanical stability, and adsorption capacity. New processing routes as in situ functionalization of membranes and 3D printing may also enable the increased and optimal utilization of nanocellulose membranes/filters in water purification.

## Figures and Tables

**Table 1:** Overview of the maximum uptake of heavy metal species by functionalized nanocellulose with a comparison to similar cellulose material, when possible.

Functionalization Nature and Route	Contaminant	pH	Q_max_ (mg/g)	Reference	Q_max_ of Comparably Functionalized Cellulose (mg/g)	Reference
None (CNF-OH obtained through mechanical treatment)	Cd^2+^	5.5	11	[46]		
	Ni^+^		11			
	Pb^+^		10			
CNC-SO^3^^−^ (sulfuric acid hydrolysis)	Ag^+^	6.5	34	[22]		
CNC-PO_4_^2^^−^ (phosphorylation of CNC)	Ag+	4	136	[48]		[67]
	Cu^2+^		117		19	
	Fe^3+^		115			
CNC-COOH (Succinic anhydride)	Pb^2+^	5.5	458	[42]		[68]
	Cd^2+^		335		179	
CNC-COOH (sodium periodate/chlorite)	Cu^2+^	4	185	[45]		
CNF-COOH (TEMPO)	Cu^2+^	6.2–6.5	112	[14,41]		
	Ni^2+^		49			
	Cr(III)		58			
	Zn^2+^		67			
	UO_2_^2+^		167			
CNF-(PO(OH)_2_)_2_	VO_3_^−^	2	194	[52]		
CNF-NH_2_ (reaction with APTES)	Ni(II)	5	179	[66]		
	Cu(II)		163			
	Cd(II)		388			
NC-ROCS_2_—Na (reaction with C2)	Cd(II)	6	154	[49]		
CNC-NH_2_ (reaction with K_2_S_2_O_8_ and ethylenediamine)	Cr(VI)		3	[19]		
BC-NH_2_ (reaction with epichlrorhydrin and diethylenetramine)	Pb^2+^	4.5	84	[47]		
	Cu^2+^		63			
CNC-NH_2_ (grafting with PEG-NH_2_)	Hg^2+^			[54]	288	[53]
CNC-CONH_2_ (grafting of isopropylacrylamide)	Hg^2+^			[56]	710	[55]
CNC-PLA (grafting)	Cu^2+^	4.9		[59,60,61]	104	[57,58]
	Ni^2+^	5.9			168	
	Cd^2+^	5.7			97	
	Pb^2+^	4.5			49.7	
TEMPO CNF-PEI	Cu^2+^	5	52.3	[63,64]	19.2	[64]
CNF-CMC (crosslinked using butanetetracarboxylic acid)	Ag^+^		106	[65]		
	Cu^2+^		74.8			
	Pb^2+^		111.5			
	Hg^2+^		131.4			

**Table 2:** Overview of the maximum uptake of organic molecules by functionalized nanocellulose with a comparison to similar cellulose material when possible.

Functionalization Nature and Route	Contaminant	pH	Q_max_ (mg/g)	Reference
CNC-SO_3_^−^ (sulfuric acid hydrolysis)	Methylene blue	9	118	[70]
	Tetracycline hydrochloride		7	[80]
CNC-COOH (TEMPO)	Methylene blue	9	769	[70]
CNC-COOH (esterification with maleic anhydrid)	Crystal violet	6	244	[71]
CNC-COOH (reaction with ammonium persulfate)	Methylene blue	7	101	[73]
CNC-COOH (reaction with citric acid)	Methylene blue	7	135	[74]
CNC-NH_2_ (oxidation with NaIO_4_ followed by reaction with ethylenediamine)	Acid red GR	4.7	556	[27]
CNF-NR^4+^ (reaction with EPTMAC)	Congo red		664	[75]
	Acid green 25		683	[75]
	Humic acid		310	[77]
CNC-NH_2_ (grafted with PVAm)	Acid red GR	9	869	[76]
	Congo red 4BS		1469	
	Light yellow K-4G		1250	
CNC-NH_2_ (oxidation with NaIO_4_ followed by grafting with hyperbranched PEI)	Congo red		2100	[77]
	Basic yellow		1860	
